# Metabolomics Combined with Sensory Analysis Reveals the Impact of Different Extraction Methods on Coffee Beverages from *Coffea arabica* and *Coffea canephora* var. Robusta

**DOI:** 10.3390/foods11060807

**Published:** 2022-03-11

**Authors:** Fosca Vezzulli, Gabriele Rocchetti, Milena Lambri, Luigi Lucini

**Affiliations:** 1 Department for Sustainable Food Process, Università Cattolica del Sacro Cuore, Via Emilia Parmense 84, 29122 Piacenza, Italy; fosca.vezzulli@unicatt.it (F.V.); milena.lambri@unicatt.it (M.L.); luigi.lucini@unicatt.it (L.L.); 2 Department of Animal Science, Food and Nutrition, Università Cattolica del Sacro Cuore, Via Emilia Parmense 84, 29122 Piacenza, Italy

**Keywords:** coffee, extraction methods, sensory analysis, metabolomics

## Abstract

An untargeted metabolomics approach combined with sensory analysis was used to depict the impact of different traditional Italian extraction methods (i.e., Espresso, Neapolitan, Moka) along with Filter, on *Coffea arabica* and *Coffea canephora* var. robusta beverages. To this aim, polyphenols, Maillard reaction products, and coffee metabolites were screened by high resolution mass spectrometry and elaborated through both unsupervised and supervised multivariate statistical approaches. Multivariate statistics showed a distinctive chemical profile for Espresso preparation, while Moka and Neapolitan were very similar. The orthogonal projection to latent structures and discriminant analysis allowed the identification of 86 compounds showing a high VIP discrimination score (i.e., > 0.8). The 2,5-dimethyl-3-(methyldithio)-furan was a marker for the Filter preparation, while 1,2-disinapoylgentiobiose characterized both Filter and Neapolitan extractions. Caffeine (known to be a bitter compound) accumulated highly in Filter vs. Espresso, although at the sensory profile, bitterness was more perceived in Espresso. Vegetal aroma carried by pyrazines, pyridines, and phenolic acids were markers of Espresso, with Robusta showing higher values than Arabica. Notwithstanding, our findings showed that the extraction process played a hierarchically higher role in driving the chemical composition of the beverages when compared to coffee species.

## 1. Introduction

Two Coffea species, namely *Coffea arabica* and *Coffea canephora* var. robusta, are the most cultivated worldwide and dominate in terms of market volume [1]. These species are deeply different genetically (polyploid for the former, diploid for the latter) [2], require different pedoclimatic conditions [3], have different biochemical ripening processes, and undergo different post-harvesting processes [4,5,6,7]. Therefore, chemical profiles and flavor precursors characterizing the green beans reflect the previously cited diversity [8,9]. Also, the transformations occurring during roasting and extraction steps can lead to exclusive metabolites, such as aromatic compounds and bioactive molecules, providing a cup profile to the beverage that testifies all those characteristics [10,11].

Together with the coffee powder used, it is recognized that the extraction method strongly impacts the sensory profile of coffee beverages [12]. Among others, the traditional Italian extraction methods, namely Moka, Neapolitan pot, and Espresso, are known to give consumers a unique and recognizable aromatic and gustative perception [13,14]. In this context, the market availability of automatic filtered coffee from domestic machines has contributed to enhancing the consumption of less intense and longer coffee, even in a historically espresso-consumer population [15]. Conversely, an increasing interest in the rediscovery of home extraction systems stressed the need for accurate studies on technical aspects [16] to exalt and differentiate the final cup from Moka Neapolitan pots [17].

In recent years, several analytical strategies have been implemented regarding the quality and integrity of foods, including coffee and coffee beverages, such as isotope ratio mass spectrometry (IRMS), liquid chromatography coupled with mass spectrometry (LC-MS), gas chromatography coupled with mass spectrometry (GC-MS), near infrared spectroscopy (NIRS), and nuclear magnetic resonance spectroscopy (NMR) [18]. In this regard, liquid chromatography quadrupole time-of-flight mass spectrometry approach has allowed discrimination against coffee brewed by different extraction methods [19]. Similarly, high-resolution mass spectrometry techniques have been efficiently applied for evaluating coffee quality and the potential correlations with the sensory attributes [13]. Interestingly, several studies on *C. canephora* have been carried out in recent years by using metabolomics [20,21]. In this regard, this species is considered to have a lower cup quality compared to *C. arabica.* Accordingly, some authors were able to identify potential markers for the early selection of *C. canephora* plants with desirable cup quality traits [21]. Therefore, metabolomic approaches demonstrated a solid potential to investigate several aspects related to coffee quality, including processing conditions (e.g., roasting, grinding, and brewing methods), authentication, traceability, the correlation with sensory quality, and the quality improvements of selected cultivars.

However, to the best of our knowledge, there is still a lack of information in the scientific literature about a link between the comprehensive metabolomic phytochemical profiles of coffee and sensory traits, related to different extraction methods. Therefore, this study aimed to explore the potential correlations existing between metabolomic profiles of *C. arabica* and *C. canephora* var. robusta beverages obtained through four traditional extraction methods (Moka, Neapolitan pot, Espresso, and Filter) and their sensory profile. This information is relevant to unravel the effect of extraction method and coffee species combinations, in terms of both sensory and chemical profiles. This piece of information can complement the more consolidated knowledge already available on other quality-related aspects such as planted cultivar and edaphic conditions, processing and roasting, as well as shelf life and packaging, in a “one-quality” perspective.

## 2. Materials and Methods

### 2.1. Coffee Samples

Two roasted coffee samples were supplied from a local industrial roaster (Musetti, Piacenza, Italy) with the same roasting process. The two commercial blends were constituted either by 100% *C. Arabica* natural processed from Brazil or 100% *C. Canephora* var. robusta natural processed from India. Coffee samples were ground with La Cimbali ELECTIVE (Gruppo Cimbali S.p.A., Binasco, Milan, Italy) grinder-doser to reach the proper granulometry for each extraction.

### 2.2. Extraction Methods

Moka extraction was performed using the Bialetti “Moka Express” as provided by [22], applying an adjusted brew ratio of 76 g/L for both Arabica and Robusta samples, for both sensory and chemical analysis. Filter coffee was prepared using a commercial drip coffee maker Ariete Vintage, setting a “strong coffee” modality, and using a brew ratio of 50 g/L. Neapolitan coffee was prepared using an aluminum traditional Neapolitan pot (Ilsa, Turin) following the procedure described by [17] using a brew ratio of 72 g/L.

Traditional Italian espresso coffee was prepared with the professional Espresso machine Cimbali M100 (Gruppo Cimbali S.p.A., Binasco, Milan, Italy) using water softened from a Brita Purity C150 (30% bypass) to obtain acceptable total and carbonate hardness, according to the SCA water control chart [23]. The extraction was made at 92 °C, with six sec of pre-infusion at a ratio between coffee powder and beverage of 1:2 (*w*/*w*). About 16 g of coffee were packed in a double shot coffee basket for both Arabica and Robusta samples.

### 2.3. Sensory Analysis

The sensory evaluation was performed by a single panel of six trained panelists in two different sessions, the former for espresso and filter coffees, the latter for Moka and Neapolitan pot extractions, both carried out in laboratory “SensoryLab”, compliant with UNI ISO 8589 standards, at Università Cattolica del Sacro Cuore (Piacenza—Italy). The attributes characterizing the samples were selected by the judges after a training session using Coffee Lexicon as reported in the literature [24]. Then, the selected attributes were listed in M34 Trialcard Plus form (Figure S1) by “Centro Studi Assaggiatori—Italian tasters”, which was used during each session to rate samples. In detail, panelists were asked to evaluate each attribute on a scale from “0” set as “absence of perception” to “9” set as “very intense perception”. The validation and replicability of each panelist were tested by the presence of one duplicated sample per session. Medians of the scores given to each descriptor from a single panelist to the duplicated samples were not to differ from more than ±1 point to consider the panelist as being repeatable. Then, before starting each session of sensory analysis, a panel calibration was made by delivering panelists with the median score reached by an extra sample of coffee beverage. Data were collected with ADS System by Horizon Design and Centro Studi Assaggiatori Brescia, to be statistically elaborated.

### 2.4. Extraction of Metabolites from Coffee Samples

For the metabolomics analysis, a total of 44 samples were analyzed, when considering Espresso (20 replicates), Neapolitan (eight replicates), Moka (eight replicates), and Filter (eight replicates) preparations. In this regard, one mL of each coffee beverage (as resulting from Moka, Neapolitan pot, Espresso, and Filter preparations) were extracted in five mL of 70% aqueous methanol (LC-MS grade, VWR, Milan, Italy) acidified with 0.1% formic acid. Regarding the starting ground coffee samples of *C. arabica* and *C. canephora* var. robusta, four replicates (1 g) of each sample were extracted using an Ultra-Turrax homogenizer (IKA T25, Staufen., Germany) using the same extraction solution. The extracts were then centrifuged (Eppendorf 5810R, Hamburg, Germany) at 10,000× *g* for 10 min at 4 °C and filtered using 0.22 μm cellulose syringe filters into amber vials.

### 2.5. Untargeted Metabolomic Profiling through UHPLC-QTOF Mass Spectrometry

The untargeted metabolomic profile of the different coffee extracts was investigated through an UHPLC-QTOF-mass spectrometry. To this aim, a 1290 liquid chromatograph was coupled with a G6550 mass spectrometer detector via a Dual Electrospray Jet Stream ionization system (from Agilent Technologies, Santa Clara, CA, USA) under previously optimized instrumental conditions [13]. The instrument worked in Full-SCAN mode, acquiring positive ions in the range of 100–1200 *m*/*z*. Samples were acquired in “extended dynamic range” mode with a nominal resolution of 40,000 FWHM. The injection volume was 6 μL, while the sequence injection was randomized. Also, Quality Control samples (QC) were injected in the UHPLC-QTOF and consisted of a pooled aliquot of each extract. In this regard, QCs were injected at the beginning of the sequence and every 10-sample injection and analyzed in data-dependent MS/MS mode using 10 precursors per cycle (1 Hz, 50–1200 *m*/*z*, positive polarity, active exclusion after two spectra), with typical collision energies of 10, 20, and 40 eV. The raw mass features were aligned and deconvoluted using the Agilent Profinder B.06 software. In this regard, the find-by-formula algorithm was used to annotate molecular features (MFs) following mass and retention time alignment. The detailed information regarding the post-acquisition process is accurately described elsewhere [13]. Three databases were combined for the identification process, namely the FoodDB (https://foodb.ca/ (accessed on 1 February 2022) using the list of compounds already reported in coffee), Phenol-Explorer 3.6 (http://phenol-explorer.eu/ (accessed on 1 February 2022) to profile polyphenols), and a custom database on Maillard reaction products. Based on our process, each compound was identified according to a Level 2 of confidence (putative annotation based on high mass accuracy, exploiting the isotopic profile of each mass feature) as reported by COSMOS Metabolomics Standards Initiative [25]. Besides, the level of confidence in annotation was increased by using the spectral information reported in the QC. The latter were elaborated upon using the software MS-DIAL (version 4.70) for a further identification and/or confirmation step [26] and compared against the publicly available MS/MS experimental spectra available in the same software (e.g., Mass Bank of North America) and MS-Finder in-silico fragmentation from compounds in Lipid Maps, FoodDB, and PlantCyc [26].

### 2.6. Multivariate Statistical Analysis

#### 2.6.1. Metabolomic Data

The raw data obtained following metabolomics were aligned and normalized using the Agilent Mass Profiler Professional B.12.06 software, according to the workflow reported in previous work [13]. Then, two different multivariate statistical approaches were used to elaborate on the raw data, namely an unsupervised hierarchical cluster analysis (both HCA-distance measure: Euclidean; clustering algorithm: Ward’s, and PCA-principal component analysis) and a supervised orthogonal partial least squares discriminant data analysis (OPLS-DA). In particular, the supervised model was created considering as class discrimination the “extraction process”. Besides, each OPLS-DA model was inspected for outliers, cross-validated (CV-ANOVA), and evaluated for potential overfitting (permutation testing with 200 random permutations). The model parameters (goodness of fit: R^2^Y and goodness of prediction: Q^2^Y) were also inspected to evaluate the overall goodness of the prediction model. The variables importance in projection (VIP) was finally used to select those compounds having the highest discrimination potential (VIP score > 0.8) and potentially related to the sensorial profile [13,27].

#### 2.6.2. Sensory Data

All the data were collected with Microsoft Excel 2007 and elaborated upon by radar graphs. Discriminant power of the extraction method was assessed via one-way analysis of variance (ANOVA). A factorial analysis with principal component analysis (PCA) elaboration was applied to highlight relationships within variables and between variables and samples, which were clustered as such for the specie and extraction method. Statistical elaboration was carried out by IBM SPSS Statistics 27 (IBM Corporation, Armonk, NY, USA).

## 3. Results and Discussion

### 3.1. Untargeted Profiling by UHPLC-QTOF-Mass Spectrometry

In this work, the untargeted metabolomics approach based on UHPLC-QTOF-mass spectrometry resulted in the putative identification of 228 compounds annotated according to a Level 2 of confidence [13,25]. Besides, the analysis of QC samples allowed us to confirm the structural identity of 94 compounds, such as caffeoylcholine, caffeine, phloroglucinol, and trigonelline, amongst others. A comprehensive list reported the relative abundance of each compound annotated, together with its composite MS and MS/MS spectra, can be found in the Supplementary Materials (Supplementary File S1).

As the first step, we used a Volcano plot analysis combining ANOVA (*p* < 0.05) and Fold-Change analysis (FC cut-off > 1.2) to check the chemical differences between the raw ground coffee samples under investigation (i.e., *C. arabica* and *C. canephora* var. robusta) before running the different extraction processes. The output obtained is reported in Supplementary File S1. As can be observed, the volcano plot for the comparison *C. arabica* vs. *C. canephora* var. robusta showed 92 significant compounds (including the isomeric structures), with 72 compounds significantly up accumulated for the *C. arabica* ground coffee, thus revealing a broader and complex phytochemical profile when compared to *C. canephora* var. robusta.

Regarding specific and typical compounds, according to [28], caffeine was strongly up accumulated in *C. canephora* var. robusta (Fold Change value = 10.81; *p*-value = 0.044), followed by Na-*p*-hydroxy-coumaroyl-tryptophan (Fold Change value = 3.10; *p*-value = 1.7 × 10^−6^), (R)-2-hydroxy-4,7-dimethoxy-2H-1,4-benzoxazin-3(4H)-one 2-glucoside (also known as HDMBOA-Glc) (Fold Change value = 2.01; *p*-value = 8.5 × 10^−6^) and coffeasterene (Fold Change value = 1.95; *p*-value = 1.2 × 10^−5^). Overall, the amino acid conjugates of hydroxycinnamic acids (e.g., Na-*p*-hydroxy-coumaroyl-tryptophan) have been previously reported as potential marker compounds to discriminate among coffee cultivars [29]. In this regard, [30] showed that *p*-coumaroyl-N-tryptophan was a characteristic marker compound of the *C. canephora* species, thus confirming our findings. Also, the compound coffeasterene belongs to the class of organic compounds known as stigmastanes and derivatives. These are sterol lipids with a structure based on the stigmastane skeleton, which consists of a cholestane moiety bearing an ethyl group at the carbon atom C24; however, little information is available in the literature concerning its ability as related to cultivar discrimination. Finally, HDMBOA-Glc has been reported as a marker of biological interest when considering defense mechanisms of the plant [31]; therefore, our findings suggested a potential up-accumulation of this metabolite in *C. canephora* var. robusta as a response to terroir-related factors, such as pedo-climatic conditions, together with agronomic and post-harvest practices [32].

Regarding the significant marker compounds of *C. arabica*, those showing the highest variations were *p*-HPEA-AC (Fold Change value = 2.13; *p*-value = 3.8 × 10^−6^), 5-methylquinoxaline (Fold Change value = 1.88; *p*-value = 1.4 × 10^−4^) and isomeric forms of cyclopentanedione (Fold Change value = 1.83; *p*-value = 5.1 × 10^−5^). The compound *p*-HPEA-AC belongs to the class of organic compounds known as tyrosols and derivatives. These antioxidant compounds are minor phenolic compounds in the coffee plant, although their presence has been previously documented [13,33]. Besides, 5-methylquinoxaline belongs to the chemical class of quinoxalines; these compounds contain a quinoxaline moiety, a bicyclic heterocycle made up of a benzene ring fused to a pyrazine ring. According to the literature, 5-methylquinoxaline contributes to the coffee flavor development, being associated with sensorial descriptors, such as burnt, roasted, nutty, and roasted corn [34]. Finally, cyclopentanedione derivatives (such as 3,5-dimethyl-1,2-cyclopentanedione), also known as benzyl-related compounds, belong to the class of organic compounds known as cyclic ketones, usually described as sweet, maple, sugar, caramel, and coffee tasting compounds, and then considered potential biomarkers for the consumption of coffee and coffee products [35].

### 3.2. Multivariate Statistical Discrimination of the Different Extraction Methods

**Figure 1:**
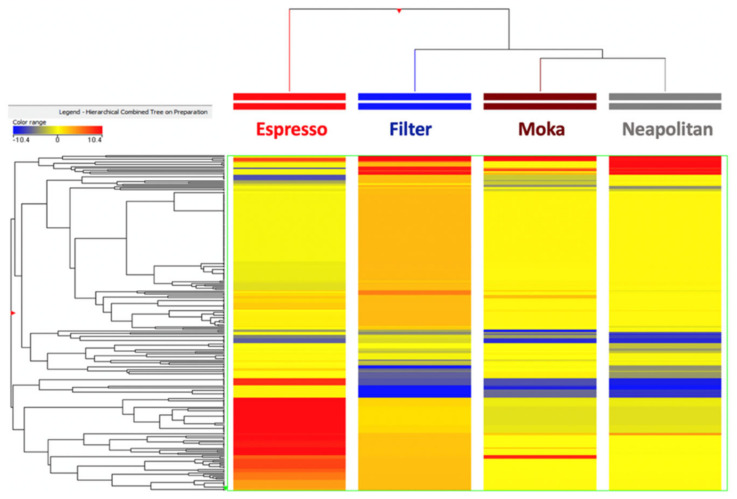
Unsupervised hierarchical cluster analysis (HCA) based on fold-change heat map (similarity: Euclidean; linkage rule: ward) for the different coffee samples included in the different extraction category (i.e., Espresso, Filter, Moka, and Neapolitan).

In the next part of this work, untargeted metabolomics based on UHPLC-QTOF mass spectrometry was used to explore the major differences imposed on the chemical profile by the four different extraction methods under investigation, thus accounting for the variability imposed specifically by each processing method. As can be observed from the unsupervised hierarchical cluster analysis heat map (Figure 1), the Espresso preparation was characterized by the most distinctive chemical profile, being included in a separate cluster. Interestingly, Filter, Moka, and Neapolitan preparations were included in another cluster, with Moka and Neapolitan providing a more similar profile, being included in the same sub-cluster.

**Figure 2:**
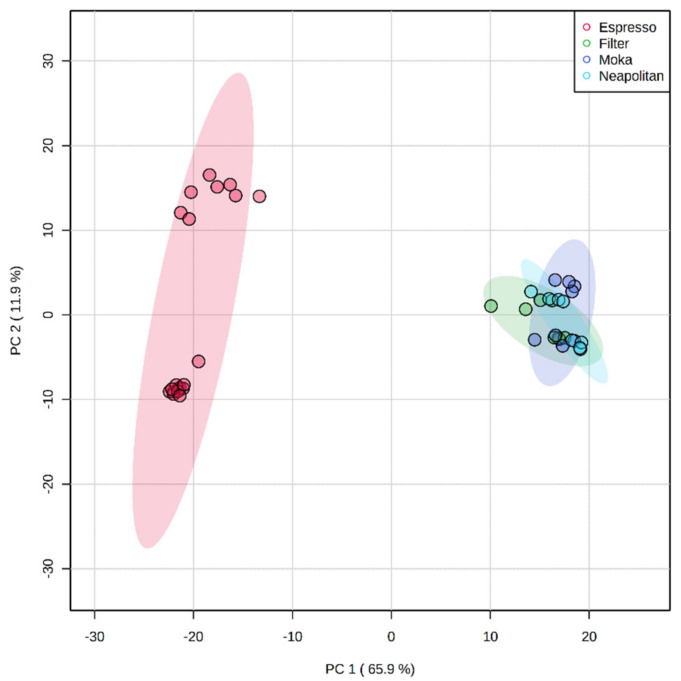
Principal Component Analysis (PCA) score plot for the different coffee samples included in the different extraction category (i.e., Espresso, Filter, Moka, and Neapolitan).

Besides, a PCA score plot was inspected to assess the dispersion of each sample according to the measured chemical profile. As clearly reported in Figure 2, the two main principal components (PC1 and PC2) were found to explain a total of 77.8% of the variability among each group, thus revealing a clear ability of the statistical model to discriminate the different extraction methods. Also, a high variability between the Espresso samples was observed, mainly driven by the different cultivars considered (i.e., *C. arabica* and *C. canephora* var. robusta).

**Figure 3:**
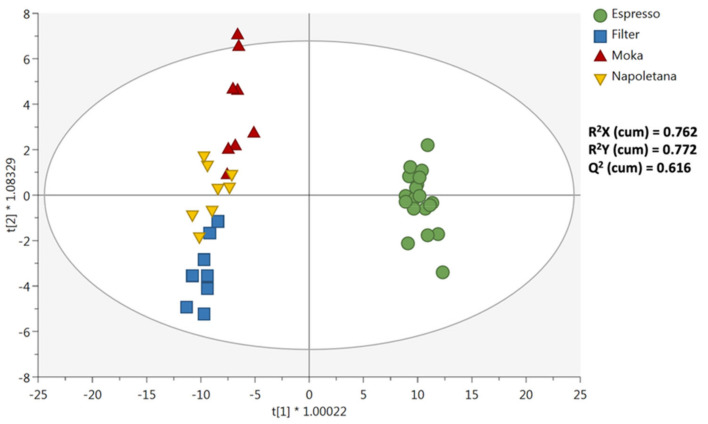
Orthogonal Projections to Latent Structures Discriminant Analysis (OPLS-DA) score plot for the different coffee samples included in the different extraction category (i.e., Espresso, Filter, Moka, and Neapolitan).

Thereafter, to better investigate the compounds or classes of compounds explaining most of the variability observed, the following supervised multivariate statistical approach, namely OPLS-DA, was used. The OPLS-DA score plot is reported in Figure 3. The goodness model parameters were highly significant, being: correlation R^2^Y (cum) = 0.772, R^2^X = 0.762, and Q^2^Y prediction ability = 0.616. Also, the prediction model was cross-validated using a Cross Validation-ANOVA (*p*-value = 2.15 × 10^−14^) and both strong outliers and overfitting could be excluded (Supplementary File S1). Besides, Figure 3 indicates that the orthogonal components were effective in separating the Espresso vs. the other extraction methods, while the chemical distance between coffee samples included in the Filter, Moka, and Neapolitan groups was smaller.

After that, the identification of the most important variables in the orthogonal projection was carried out through the VIP method. This approach ranked compounds as a function of their ability to determine the OPLS-DA score plot observed in Figure 3. These discriminant compounds are reported in Table 1, together with their VIP scores (cut-off > 0.8) and Log2 Fold-Change values (resulting from Fold-Change analysis with cut-off = 1.2 and having a *p* value < 0.05). The Espresso category was used as reference in Fold-Change analysis. Overall, we classified 86 discriminant compounds (excluding the potential isomeric structures), showing large differences between the different coffee samples extracted with the four extraction methods. Among the discriminant compounds, we found a large abundance of polyphenols (42%), followed by amino acids analogues, pyrazines, pyridines, and aryl-alkyl-ketones. Overall, two compounds were characterized by the highest VIP scores, namely 2,5-dimethyl-3-(methyldithio)-furan (VIP score = 1.72) and 1,2-disinapoylgentiobiose (VIP score = 1.48), belonging to furan derivatives and phenolic acids classes, respectively. Interestingly, these latter were highly discriminant for the Filter preparation, as can be observed by checking the LogFC variations reported in Table 1.

Ubiquitously present in thermally processed foods, furans exposure studies revealed that coffee contributes most significantly to an adult’s dietary exposure. This aspect might be of concern, considering that the International Agency for Research on Cancer classified furan as type 2B (i.e., possibly carcinogenic to humans). Besides, coffee is one of the only foods known where 2-methylfuran levels consistently exceed those of furan. However, as [36] reported, methyl-furans appear to be metabolized, at least in part, in a similar manner to furan, thus resulting in highly reactive intermediates with similar toxicity. Regarding their presence in coffee beverages, initially absent in green coffee beans, furan derivatives are generated upon roasting from the thermal degradation of endogenous components. As reported by [37], methyl-furan forms are generated from the condensation of carbohydrate moieties arising from the Maillard reaction, while the origins of 3-methyl-, 2,5-dimethyl-, and 2,3-dimethyl-furan derivatives have yet to be fully established [38]. Besides, 1,2-Disinapoylgentiobiose is a phenolic acid that belongs to the sub-class of hydroxycinnamic acids. Coffee is known to be a rich source of polyphenols, especially hydroxycinnamic acids, such as different isomers of caffeoylquinic acid [39]. In our experimental conditions, this compound was highly abundant in Filter (LogFC = 9.30) and Neapolitan (LogFC = 9.01) extractions when compared with Espresso (Table 1). On the other hand, we found that the Espresso category was the best in providing the highest recovery of chlorogenic acid isomers (Table 1), with the Filter and Neapolitan category characterized by a strong down-accumulation for these compounds (on average: −8.44; Table 1). Looking at other typical compounds, we found that Filter was the best extraction system for the recovery of caffeine (VIP score = 1.01; LogFC vs. Espresso = 3.44), while the group of pyrazines mainly characterized the Espresso preparation with the families of 2-Acetyl-dimethyl-pyrazines showing the highest discrimination potential (VIP score = 1.11). According to data from the literature, pyrazines and furans are the major compounds in terms of concentration and the main classes contributing to coffee characteristic aroma through their impact on flavor, imparting earthy, musty woody, and papery notes. In previous work, [40] identified 12 pyrazines in different brands of capsule-brewed Espresso samples, with a significant abundance of 2-ethylpyrazine, 2-ethyl-6-methylpyrazine, and 2-ethyl-3,5-dimethylpyrazine, that have also been indicated as potent key odorants. In our untargeted experimental conditions, we detected several isomeric forms of different pyrazine-derivatives belonging to ethyl-, acetyl-, diethyl-, and dimethyl-derivatives (Table 1). Therefore, the distribution of pyrazines demonstrated that Espresso preparation was the best in enhancing the potential development of typical coffee aroma.

Looking at some recent works about coffee brewing, [41] evaluated the distribution of α-dicarbonyl compounds (α-DCs) and 4-methylimidazole in 72 Espresso coffees made with different roasting and brewing conditions, demonstrating that a cold brewing method provides the maximum concentration of these potentially hazardous compounds when the largest coffee bean particles were used. Moreover, the level of α-DCs was higher in *C. arabica* than in *C. robusta*, while *C. robusta* showed higher levels of 4-MI when compared with C. arabica. In our experimental conditions (UHPLC-QTOF-MS), we did not evaluate the presence of these Maillard reaction/caramelization-related intermediates considering that the untargeted full Scan acquisition ranged from 100 up to 1200 *m*/*z*. However, as showed in Table 1, among the discriminant compounds we listed several isomeric forms of methylated and dimethylated oxazoles. Interestingly, the formation of the different heterocyclic volatile compounds in coffee represents a complex interplay involving several chemical reactions, such as the so-called Strecker degradation, in which the dicarbonyl reagent undergoes transamination, thus leading to an α-aminocarbonyl. The α-aminocarbonyls are not only the precursors of pyrazines but can also lead to pyrrole derivatives (some of them included among the best discriminant marker compounds; Table 1), as well as imidazole and oxazole derivatives (for the latter, a shared and parallel formation mechanism has been previously proposed by [42]. Our findings revealed that both pyrrole and oxazole derivatives were marker compounds of the Espresso preparation, thus confirming once again the most complex chemical profile as potentially related to its typical aroma.

### 3.3. Sensory Analysis

In this work, all the panelists properly performed in terms of repeatability; therefore, the raw data generated by the six panelists have been validated and provided in Table 2 as the median values of the panel score for each sensory descriptor for each sample under investigation. Interestingly, the sensory evaluation was more relevant in discriminating the different extraction methods than the coffee species.

The analysis of variance (ANOVA) provided the classification of samples by significantly variant descriptors, namely color intensity, aroma intensity, body, acidity, vegetal, stone fruit, nuts and dry fruits, caramel, cocoa, burnt, positive aromas, and aroma persistence. Espresso samples were highlighted as the most intense (*p* < 0.05) in terms of aroma, acidity, body, and aroma persistence, being characterized in terms of caramel and stone fruits notes, the most persistent for aroma, and the weakest for burnt notes.

On the other hand, Neapolitan pot resulted as the weakest in color and aroma intensity, acidity, stone fruits notes, yet was the most characterized for cocoa notes. Regarding Moka and filter coffee, they hardly never peaked in any category apart from burnt notes, which was higher in Moka. Body, vegetal, nuts, and dried fruits, caramel, cocoa, and aroma persistence resulted in the weakest intensity in filter coffee. Additionally, Figure 3 provides the sample distribution in two dimensions, rotate space drove, from PCA on average data obtained by the sensory analysis of different coffee beverages corresponding to four extraction systems applied on Arabica and Robusta roasted coffee.

**Figure 4:**
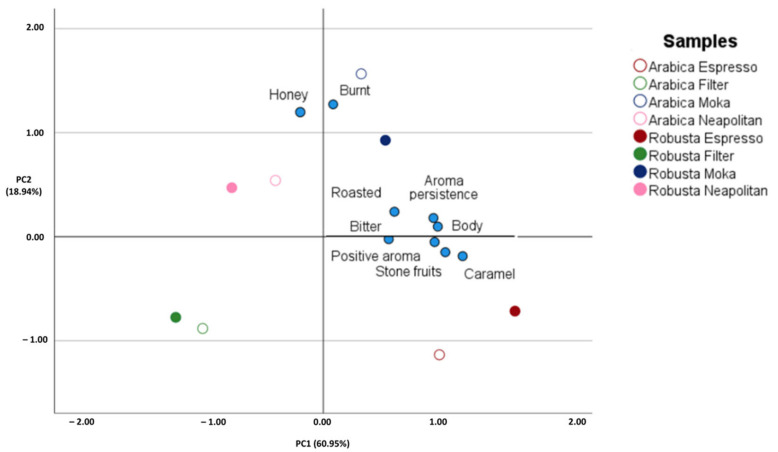
Unsupervised PCA score plot resulted from data obtained by sensory analysis of different coffee beverage corresponding to four extraction systems applied on Arabica and Robusta roasted coffee.

The PCA graph (Figure 4) showed the different sensory perceptions (i.e., bitter taste, body, aroma persistence, burnt, caramel, honey, roasted, stone fruits and global positive aroma) that led to discrimination among samples depending on coffee variety and/or extraction methods. The two principal components (PC1 and PC2) cumulatively explained the 79.9% of the total variance, thus outlining the reliability of sensory attributes to discriminate against samples based on either the origin or further handling. Additionally, as outlined in Figure 4, such factorial analysis applied to the sensory data of coffee beverages showed a higher impact of the coffee preparation method over coffee variety in providing samples grouping.

**Figure 5:**
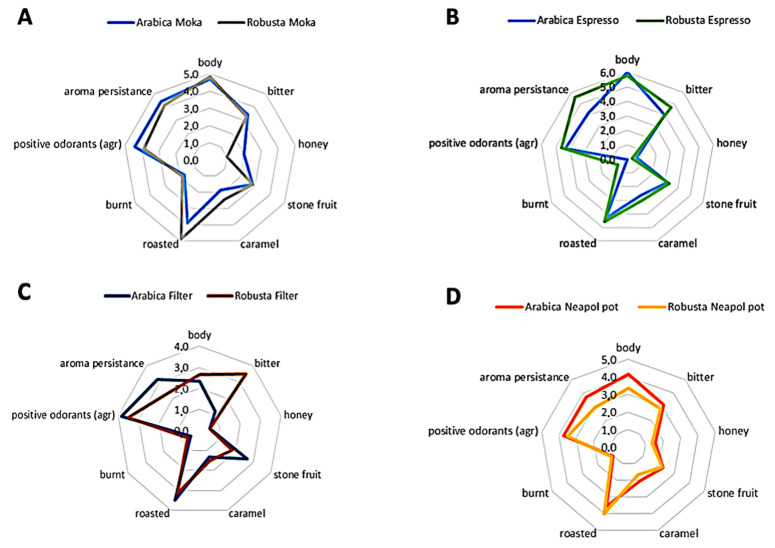
Sensory profiles of different coffee beverages corresponding to four extraction systems applied on Arabica and Robusta roasted coffee, and considering: Moka (**A**), Espresso (**B**), Filter (**C**), and Neapolitan (**D**) extraction methods.

Furthermore, by inspecting the distribution in Figure 5A–D, it was possible to define the most discriminant descriptors of different extraction. In this regard, the Moka brewing (Figure 5A) was found to exalt body, roasty, and caramel aromas for Robusta, however, it was relevant for the extraction of the positive odorants and honey notes characterizing Moka coffee obtained from Arabica, while increasing their persistency. This also reflected the high relative abundance of 2-acetylpyrrole (caramel, bread and beaked) and 4,5-dimethyl-2-propyloxazole (roasted) in Arabica that justify the cereal and pastry taste and the higher presence in Robusta of 3-Ethylpyridine (grassy) and 6-Acetyl-2,3,4,5-tetrahydropyridine (creamy, bread crust) (Table 1).

When considering the Espresso extraction (Figure 5B), Arabica overcame Robusta thanks to the higher body, the richness in positive aroma and fragrances and the pastry notes. Conversely, Robusta showed higher bitterness and aroma persistence, together with aromas of caramel, roasted and stone fruits. As confirmed by the shape of the spider graphs, the PCA and the cluster analysis of metabolites Espresso extraction were closer in terms of perceptions and composition than the other extractions prepared with the same roasted coffee.

Regarding filter coffee prepared with an automatic home dripper (Figure 5C), it was characterized by constant descriptors reported for both Arabica and Robusta, and it was accepted that the bitterness that was peculiar only for the drip-coffee obtained with Robusta beans. Two samples differed for the predominance of roasted, stone fruits notes and a more intense olfactory perception always found in Arabica coffee. Conversely, Robusta was found to provide more caramelized notes—in terms of quantity—and higher body.

Finally, the Neapolitan pot (Figure 5D) remarkably impacted the profile of the beverage. In fact, as highlighted from the slight distances of samples both in the PCA graph and on the spider chart, the two species were lower in scores for all the descriptors if compared with other extraction methods and were mutually close in terms of sensory profile. The only notable differences revealed by the panelists were body and aroma persistence, majorly perceived in the Arabica sample, and roasted and burnt notes higher in Robusta coffee.

## 4. Conclusions

The combination of UHPLC-QTOF untargeted metabolomics and sensory analysis allowed us to depict the impact of different Italian traditional extraction methods, namely Espresso, Neapolitan, Moka, and Filter coffee. The chemical and sensory profile of each coffee beverage was evaluated using both *Coffea arabica* and *Coffea canephora* var. robusta. Interestingly, the ability of our approach to discriminate against coffee beverages, prepared with different methods, was hierarchically higher than the coffee species considered. The combination of sensory analysis and metabolomics allowed us to build distinctive profiles characterizing brewed coffees, thus outlining mutual differences and similarities. Further ad hoc studies, based on more targeted approaches, are advisable to better evaluate the degree of correlation between sensory perceptions and chemical markers as a function of the extraction technique considered.

## Figures and Tables

**Table 1:** Classified VIP discriminant compounds (VIP score > 0.8) following the OPLS-DA supervised statistics and considering the comparisons of Filter, Moka, and Neapolitan vs the Espresso extraction system, according to the Log2 Fold-Change (FC) variations.

Class	Discriminant Compounds (OPLS-DA)	VIP Score	LogFC Filter vs. Espresso	LogFC Moka vs. Espresso	LogFC Neapolitan vs. Espresso
Alkaloids	Caffeine	1.01	3.44	−0.12	−2.48
	Calystegine A6	0.99	0.74	−0.99	−1.96
Alkyl-phenylketones	3′,4′-dihydroxyacetophenone	1.26	3.78	1.62	1.47
	1-phenyl-1-propanone	1.10	−16.45	−18.91	−18.98
Amino acids, peptides, and analogues	L-homoserine	1.07	1.66	−0.25	−0.50
	N-(carboxymethyl)lysine	1.04	−3.50	−1.20	−1.61
	N6-formyl lysine	1.03	0.27	0.34	−0.03
	N-(carboxyethyl)lysine	0.91	−13.47	−15.97	−15.99
	N6-acetyl lysine	0.89	−4.13	−6.44	−6.65
	N-caffeoyltryptophan	0.81	4.74	2.54	2.34
Aryl-compounds	1-methyl-2-carboxaldehyde pyrrole/2-acetylpyrrole	1.37	3.25	1.29	1.20
	1-(2-furanyl)-1-butanone	1.37	0.10	0.53	0.20
	2-acetyl-6-methylpyridine/2-acetyl-5-methylpyridine	1.09	−0.53	−0.22	−0.52
	4-acetyl-3-methylpyridine/4-acetyl-2-methylpyridine	1.09	−0.52	−0.21	−0.52
	Ethyl 2-furanyl diketone	0.90	3.89	1.58	1.49
	1-(5-methyl-2-furanyl)-1,2-propanedione	0.82	3.87	1.56	1.47
Azoles	5-ethyl-2-methyloxazole/5-ethyl-4-methyloxazole/4-ethyl-2-methyloxazole/2-ethyl-5-methyloxazole/2-ethyl-4-methyloxazole	1.07	−15.21	−17.68	−17.73
	4-ethyl-2,5-dimethyloxazole/5-methyl-2-propyloxazole/5-ethyl-2,4-dimethyloxazole	0.97	0.18	−0.01	−0.29
	4,5-dimethyl-2-propyloxazole	0.88	2.06	−0.46	−0.93
Flavonoids	Narirutin 4′-*O*-glucoside	1.08	−4.06	2.30	−6.58
	Neodiosmin/Diosmin	1.07	3.51	0.97	1.02
	Neohesperidin/Hesperidin Quercetin 3-*O*-galactoside 7-*O*-rhamnoside/Kaempferol 3-*O*-sophoroside/Quercetin 3-*O*-rutinoside/Quercetin 3-*O*-rhamnosyl-galactoside/Kaempferol 3,7-*O*-diglucoside	1.03	3.54	1.01	1.03
	Pigment A/Peonidin 3-*O*-rutinoside/Peonidin 3-*O*-(6″-*p*-coumaroyl-glucoside)	1.03	3.52	0.99	1.01
	Delphinidin 3-*O*-rutinoside/Cyanidin 3,5-*O*-diglucoside/Cyanidin 3-*O*-sophoroside	1.03	3.62	1.06	1.08
	Cyanidin 3-*O*-glucosyl-rutinoside	0.96	17.55	12.95	15.08
	(+)-Catechin/(-)-Epicatechin	0.96	19.18	−1.36	−1.36
	Nepetin/Isorhamnetin/Rhamnetin	0.91	4.11	5.53	5.89
Furans	Dihydroactinidiolide	1.15	−6.14	−2.05	−0.21
	2,5-Dimethyl-3-(methyldithio)furan	1.72	3.27	−2.17	−4.47
	(R)-roemerine	1.06	−14.13	−8.48	−14.04
	4-[(2-Furanylmethyl)thio]-2-pentanone	0.95	−11.56	−14.05	−14.06
	2-Ethyl-4,5-dimethyloxazole	0.94	0.15	−0.03	−0.31
Other phenolics	Tyrosol/4-ethyl-1,2-benzenediol/3-ethyl-1,2-benzenediol/4-ethylcatechol	1.37	0.10	0.53	0.20
	4-hydroxyphenylacetic acid	1.17	3.89	1.74	1.55
	Sinapaldehyde	1.07	−11.90	−0.47	−4.80
	Hydroxytyrosol	0.99	2.62	0.57	0.38
	*p*-HPEA-AC	0.99	2.34	0.24	−4.35
	threo-syringoylglycerol/erythro-syringoylglycerol	0.86	2.73	0.48	0.21
	Epirosmanol/Rosmanol	0.85	−4.83	−7.10	−4.08
	Umbelliferone/4-hydroxycoumarin	0.85	9.67	4.94	6.94
	3,4-dihydroxyphenylacetic acid	0.83	0.72	0.45	0.41
	*p*-HPEA-EA/Ligstroside-aglycone	0.81	4.72	2.53	2.32
	Vanillin	0.80	3.87	1.56	1.47
	Dimethylmatairesinol	1.34	17.81	0.63	13.29
	Leonuriside A	1.07	2.04	−0.28	2.20
Pyrazines	2-acetyl-3,6-dimethylpyrazine/2-acetyl-3,5-dimethylpyrazine	1.11	−0.46	−0.02	−0.52
	Ethylpyrazine/2-ethylpyrazine/2,5-dimethylpyrazine/2,6-dimethylpyrazine/dimethylpyrazine/2,3-dimethylpyrazine	1.08	−15.30	−17.61	−17.96
	2-isopropyl-6-methoxypyrazine/2-isopropyl-5-methoxypyrazine	1.01	−5.01	−0.38	−0.76
	2-methyl-3-(2-methylpropyl)pyrazine	0.85	−2.83	−5.32	−5.35
	2,5-diethyl-3-methylpyrazine	0.84	−2.76	−5.26	−5.29
	2,3-diethyl-5-methylpyrazine	0.84	−2.81	−5.31	−5.33
	3,5-diethyl-2-methylpyrazine	0.83	−2.79	−5.29	−5.31
Pyridines	6-acetyl-2,3,4,5-tetrahydropyridine	0.98	0.20	−0.06	−0.29
	3-ethyl-pyridine	0.89	0.35	0.12	−0.15
	2-ethyl-5-methylpyridine	0.88	2.08	−0.45	−0.92
Pyrroles	N-furfurylpyrrole/1-furfurylpyrrole	0.89	−14.44	−16.93	−16.96
	1-(2-furanylmethyl)-1H-pyrrole	0.82	−14.45	−16.93	−16.97
	2-acetyl-1-pyrroline	1.07	−15.21	−17.68	−17.73
Stilbenes	Pinosylvin	1.15	7.24	0.94	4.90
	4-vinylsyringol	1.10	−14.94	−17.44	−17.46
	Pterostilbene	0.94	−14.20	−16.69	−16.72
Phenolic acids	1,2-disinapoylgentiobiose	1.48	9.30	0.63	9.01
	Gallic aldehyde/2,4-dihydroxybenzoic acid/Protocatechuic acid/3,5-dihydroxybenzoic acid/2,6-dihydroxybenzoic acid/2,3-dihydroxybenzoic acid/Gentisic acid	1.11	−14.46	−16.95	−16.98
	5-caffeoylquinic acid/3-caffeoylquinic acid/Cryptochlorogenic acid/4-caffeoylquinic acid/1-*O*-caffeoylquinic acid/trans-neochlorogenic acid	1.07	−8.44	−0.33	−8.44
	*p*-coumaric acid ethyl ester	1.07	−4.10	2.16	−6.62
	1-sinapoyl-2-feruloylgentiobiose	1.07	−4.06	2.30	−6.59
	Caffeic acid ethyl ester	1.07	−11.90	−0.47	−4.80
	*p*-coumaroyl tartaric acid	0.95	−8.69	−11.19	−11.21
	*m*-coumaric acid/*o*-coumaric acid	0.90	−15.13	−17.44	−17.65
	Caffeic acid/trans-caffeic acid	0.85	9.67	4.94	6.94
	Vanillic acid	0.83	0.72	0.45	0.41
	4,5-dicaffeoylquinic acid/3,4-dicaffeoylquinic acid/3,5-di-*O*-caffeoylquinic acid/3,5-dicaffeoylquinic acid/4,5-di-*O*-caffeoylquinic acid	0.80	0.67	−1.83	−1.85
Other compounds	Floribundine	1.07	−13.84	−16.34	−16.37
	2-methylbenzaldehyde/4-methylbenzaldehyde/3-methylbenzaldehyde/phenylacetaldehyde/4-vinylphenol	1.05	−14.37	−6.53	−8.65
	3,5-dimethyl-1,2-cyclopentanedione/3-ethyl-1,2-cyclopentanedione/3,4-dimethyl-1,2-cyclopentanedione/3-methyl-1,2-cyclohexanedione	1.10	−13.81	−15.96	−17.06
	Damascenone	0.92	−2.38	−0.05	−0.32
	(R)-2-hydroxy-4,7-dimethoxy-2H-1,4-benzoxazin-3(4H)-one 2-glucoside	0.90	2.90	0.29	0.15
	Rubrofusarin 6-[glucosyl-(1-3)-glucosyl-(1-6)-glucoside]	0.96	16.79	12.16	16.38
	b-D-glucuronopyranosyl-(1-3)-a-D-galacturonopyranosyl-(1-2)-L-rhamnose	0.80	0.67	−1.83	−1.85
	5-methylquinoxaline	1.09	−14.22	−16.72	−16.74
	*O*-methylcorypalline	1.06	2.30	−0.05	−0.34
	3-mercapto-3-methyl-1-butanol/4-(methylthio)-1-butanol	1.04	−14.31	−6.46	−8.59

**Table 2:** Median values of sensory descriptors for different coffee beverages corresponding to four extraction systems applied on Arabica and Robusta roasted coffee. The meaning of each descriptor was explained to the panelist according to World Coffee Research [24].

Sensory Descriptors	Arabica Moka	Arabica Neapolitan Pot	Robusta Moka	Robusta Neapolitan Pot	Arabica Espresso	Robusta Espresso	Arabica Filter	Robusta Filter
Color intensity	7.0	6.7	6.9	6.1	7.0	7.3	7.0	7.2
Aroma intensity	5.4	4.1	5.6	4.0	6.7	6.2	4.8	5.2
Body	4.7	4.1	4.9	3.4	6.0	5.8	2.3	2.7
Acidity	3.1	1.4	1.9	2.1	3.8	3.3	1.8	2.2
Bitter	3.4	3.1	3.3	2.9	4.0	4.7	1.2	3.5
Astringency	2.1	2.3	1.7	1.4	2.0	2.8	1.0	1.3
Honey	2.0	1.6	1.0	1.4	0.7	0.3	0.5	0.5
Floral and fruity	2.0	1.3	1.3	1.6	1.3	0.7	1.5	1.0
Dry vegetal	2.3	2.7	3.9	2.4	2.2	3.8	2.0	1.8
Vegetal	2.7	3.1	3.9	2.9	2.3	3.1	2.0	1.7
Stone fruit	2.9	2.3	2.9	2.2	3.2	3.3	2.7	1.8
Nuts and dry fruits	3.9	3.3	4.0	3.1	2.7	3.3	2.8	2.2
Cereals	2.9	2.6	3.9	3.1	3.5	3.4	3.7	2.5
Caramel	1.9	2.0	2.4	1.6	2.7	3.0	1.3	1.5
Cocoa	2.3	2.4	2.3	2.4	1.5	2.7	0.7	1.2
Pastry	2.6	2.0	1.0	1.8	2.5	1.6	1.5	1.0
Roasted	3.9	3.6	4.9	4.0	4.3	4.6	3.5	3.2
Burnt	1.7	1.0	1.9	1.1	0.0	0.8	0.5	0.7
Positive aromas	4.4	3.7	3.9	3.5	4.3	4.6	3.8	3.5
Aroma persistence	4.4	3.7	4.1	2.9	4.2	5.6	3.2	2.3

## Data Availability

Research data are available in article supplementary materials and from the authors on request.

## Supplementary Materials

The following supporting information can be downloaded at: https://www.mdpi.com/article/10.3390/foods11060807/s1, Figure S1: Sensory attributes evaluated by the judges after description of their definition, provided as Trialcard Plus form by “Centro Studi Assaggiatori—Italian tasters”. Supplementary File S1: Dataset of coffee metabolites resulting from UHPLC-QTOF putative annotation together with the Volcano Plot analysis for the comparison Arabica vs. Robusta, and the validation parameters of the prediction model built.
