# Supplementary figures and images for: Metabolomics Combined with Sensory Analysis Reveals the Impact of Different Extraction Methods on Coffee Beverages from Coffea arabica and Coffea canephora var. Robusta
Source: Foods. 2022 Mar 11;11(6):807. doi: 10.3390/foods11060807 (PMC8953325; doi:10.3390/foods11060807)

|          |      |         |                                                                                                                                 |
|----------|------|---------|---------------------------------------------------------------------------------------------------------------------------------|
| Prodotto | Data | Giudice | 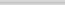 Trialcard Plus - Caffè espresso<br>16.06.2016 |
|----------|------|---------|---------------------------------------------------------------------------------------------------------------------------------|

[illegible]

Supplement: Supplementary file 1 [file foods-11-00807-s001.zip › Figure S1.pdf]
